# Cytokine-mediated pathophysiology of equine and human joint disease: mechanisms, biomarkers, and therapeutic targets

**DOI:** 10.3389/fvets.2026.1886084

**Published:** 2026-07-17

**Authors:** Tatum B. Nelson, George L. Elane, Emily Barnhardt, Kallie J. Hobbs

**Affiliations:** 1 Department of Large Animal Clinical Sciences, College of Veterinary Medicine, Texas A&M University, College Station, TX, United States; 2 Department of Veterinary Physiology and Pharmacology, College of Veterinary Medicine, Texas A&M University, College Station, TX, United States

**Keywords:** cytokines, equine, lameness, osteoarthritis, septic arthritis, synovitis

## Abstract

Cytokine-mediated inflammation plays a central role in the pathogenesis of joint disease across species, contributing to synovial activation, cartilage degradation, and structural remodeling. In both equine and human medicine, key pro-inflammatory cytokines, including interleukin (IL)-1β, tumor necrosis factor-*α* (TNF-α), and IL-6, regulate intracellular signaling pathways such as NF-κB and JAK/STAT, driving matrix degradation and immune cell recruitment. This review synthesizes current knowledge on cytokine signaling in osteoarthritis, synovitis, and septic arthritis, highlighting conserved mechanisms and species-specific differences. Comparative evaluation reveals that while cytokine profiles are largely conserved, differences in magnitude and temporal dynamics influence disease progression and therapeutic response. Emerging diagnostic applications, including synovial fluid cytokine profiling, offer potential for early detection and prognostication. Therapeutic strategies targeting cytokines, including biologics, orthobiologics, gene therapy, and mesenchymal stromal cells, demonstrate translational relevance across species. Despite advances, limitations in assay standardization and longitudinal data remain significant barriers, particularly in equine research. Strengthening cross-species methodological alignment and integrating multi-omics approaches will be critical for advancing cytokine-targeted therapies and improving outcomes in joint disease.

## 1 Introduction

Synovial joints are complex, multifunctional organs composed of articulating bone ends covered by smooth articular cartilage, a fibrous joint capsule, and supporting ligaments (1). The synovial membrane lining the capsule plays a central regulatory role in joint homeostasis, producing synovial fluid and mediating nutrient exchange through a combination of passive diffusion and synoviocyte-driven transport (2). In horses, these tissues are highly specialized to undergo high-speed locomotion and repetitive load-bearing, particularly in joints such as the metacarpophalangeal (fetlock), tibiotarsal (hock), and femorotibial (stifle) that sustain substantial cyclical stress during athletic activity (3). Mechanical forces alter cartilage permeability and fluid dynamics within the joint, influencing lubrication, nutrient delivery, and the cartilage’s ability to dissipate load (2).

Joint disease represents one of the most significant causes of lameness and economic loss in the equine industry, with osteoarthritis (OA) accounting for over 60% of clinical lameness cases (3). Equine OA is a progressive, multi-tissue disease characterized by articular cartilage degeneration, subchondral bone remodeling, and synovial inflammation, mirroring its pathophysiology in humans (4). In human medicine, OA is increasingly recognized as an inflammatory disorder, with synovitis strongly associated with pain severity and disease progression (5).

Cytokine-driven inflammation also plays a pivotal role in septic arthritis across species in both adults and neonates. In foals, hematogenous spread secondary to neonatal sepsis leads to joint infection in 14–38% of cases, with risk amplified by failure of passive transfer and other perinatal factors (6). Similar mechanisms underlie pediatric septic arthritis in humans, where metaphyseal vascular anatomy predisposes growing joints to bacterial invasion, most commonly from *Staphylococcus aureus*, *Kingella kingae*, or *Escherichia coli*, depending on age (7–9).

Despite mechanistic parallels, a substantial translational gap persists between equine and human joint research. Human studies benefit from established cytokine panels, validated clinical scoring systems, and large longitudinal cohorts, whereas equine investigations often rely on smaller sample sizes, acute synovitis models, and limited biomarker standardization (3, 10). Differences in biomechanics, athletic demands, and outcome measures further complicate cross-species comparisons. Nonetheless, key inflammatory mediators, including IL-1β, TNF-*α*, and IL-6, show consistent pathogenic roles in both species (11).

Although inflammatory joint disease is also well characterized in other veterinary species, particularly dogs, the equine-human comparison offers unique translational value. Horses develop naturally occurring osteoarthritis and athletic joint injuries that share important clinical, biomechanical, and pathological features with human disease, including progressive cartilage degeneration, synovitis, and cytokine-mediated tissue remodeling. Furthermore, human medicine has generated extensive molecular, diagnostic, and therapeutic data that can inform equine research while simultaneously benefiting from insights gained through naturally occurring equine disease models. As a result, comparative evaluation between horses and humans provides a useful framework for understanding conserved inflammatory pathways and identifying opportunities for bidirectional translational advancement.

Given these shared pathways and ongoing translational limitations, a comparative evaluation of cytokine-mediated joint pathology is both timely and necessary. This review synthesizes current knowledge on inflammatory cytokines in equine and human joint disease, emphasizing mechanistic overlap, species-specific distinctions, and emerging therapeutic and diagnostic strategies. Strengthening methodological harmonization across species will be essential for advancing our understanding of cytokine-driven joint pathology and improving outcomes for both equine and human patients.

## 2 Methods

A structured literature search was performed to identify studies evaluating cytokine-mediated mechanisms, biomarkers, and therapeutic targets in equine and human joint disease. Electronic databases including PubMed, Google Scholar, Web of Science, and Scopus were searched for relevant literature published through December, 2025. Search terms were developed to capture both species and key disease processes and included combinations of the following keywords: *“cytokines,” “osteoarthritis,” “synovitis,” “septic arthritis,” “equine,” “horse,” “human,” “interleukin,” “TNF-α,” and “IL-6.”* Boolean operators (“AND,” “OR”) were used to refine search results (Supplementary Table S1).

## 3 Sources of cytokines in joints

### 3.1 Synoviocytes

The joint capsule in both equine and human synovial joints consists of an outer fibrous layer that provides mechanical stability and an inner synovial membrane that maintains the intra-articular environment (12, 13). The synovial membrane regulates joint homeostasis and plays a central role in both normal physiology and disease processes (14, 15). Embedded within this membrane are synoviocytes, specialized cells responsible for producing synovial fluid components and modulating inflammatory activity to support functional joint movement (14).

In both humans and equids, synoviocytes synthesize key constituents of synovial fluid, including hyaluronic acid and lubricating glycoproteins such as lubricin. These glycoproteins are responsible for minimizing friction, distributing load, and supporting cartilage nutrition (15, 16). Synoviocytes also participate in the clearance of debris and soluble mediators from the joint space and regulate molecular exchange between synovial fluid and the bloodstream (14). Although their importance in maintaining joint health is well established, aspects of synoviocyte phenotypic diversity and their responses to injury, inflammation, and infection remain incompletely characterized in both equine and human medicine (15, 17).

Two principal synoviocyte subtypes have been identified: macrophage-like type a cells and fibroblast-like type B cells (18). Type A synoviocytes are motile, phagocytic cells that remove cellular debris, immune complexes, and cartilage fragments from the joint cavity. They also participate in antigen presentation and contribute to immune surveillance within the synovium (14). These cells originate from circulating mononuclear precursors and function similarly to other tissue-resident macrophages. In both equine and human inflammatory joint diseases, activation of type a synoviocytes is associated with increased production of IL-1β, IL-6, TNF *α* and matrix-degrading enzymes (3, 15).

In contrast, type B synoviocytes are fibroblast-like cells characterized by abundant rough endoplasmic reticulum, reflecting their high secretory activity. They extend cytoplasmic processes that form an organized lining along the inner surface of the synovial membrane. In both horses and humans, type B synoviocytes are primarily responsible for producing hyaluronan and other extracellular matrix components of synovial fluid (15, 19). During conditions such as OA and septic arthritis, these cells contribute to altered synovial fluid composition and participate in the production of inflammatory mediators, further influencing disease progression (3). Together, type A and type B synoviocytes coordinate immune regulation, synovial fluid production, and tissue maintenance within the joint. Dysregulation of either population contributes to synovial inflammation, altered fluid composition, and progressive joint pathology in both equine and human disease, underscoring the translational relevance of synovial biology across species (15).

### 3.2 Chondrocytes

Articular cartilage is a highly specialized, avascular connective tissue that covers the ends of bones within synovial joints in both humans and horses. It is composed of a dense extracellular matrix (ECM) organized into distinct zones, including the superficial (tangential), middle (transitional), deep (radial), and calcified cartilage layers; adjacent to this, the epiphyseal and metaphyseal regions encompass the growth plate, which is further divided into the resting, proliferative, hypertrophic, calcification, and ossification zones (20–22). Each of these contribute to load distribution, resistance to shear and compression, and maintenance of a low-friction articulating surface (21, 22). This zonal architecture is especially significant in weight-bearing joints such as the human knee and hip and the high-motion equine fetlock, hock, and stifle.

Chondrocytes are the sole resident cell type within articular cartilage and are responsible for synthesizing, organizing, and remodeling the ECM. They produce type II collagen, which provides tensile strength, and large aggregating proteoglycans such as aggrecan, which retain water and generate the osmotic swelling pressure necessary to resist compressive loads (22, 23). Through tightly regulated anabolic and catabolic processes, chondrocytes preserve the structural integrity and biomechanical properties required for normal joint function in both species.

Because articular cartilage is avascular and aneural, nutrient delivery and waste removal depend primarily on diffusion from synovial fluid. Cyclic mechanical loading during joint movement enhances fluid exchange within the matrix, facilitating metabolic support of chondrocytes (22, 24). This close functional integration between synovial fluid and articular cartilage highlights the extent to which alterations in synovial fluid composition can directly modulate chondrocyte function and viability, underscoring its central role in the maintenance of joint homeostasis.

Chondrocytes are highly sensitive and respond dynamically to mechanical stimuli, inflammatory mediators, and tissue injury. Under physiologic loading conditions, they promote matrix synthesis and tissue maintenance. However, excessive mechanical stress, IL-1β and TNF-*α* exposure, or infectious insult can induce a phenotypic shift toward catabolic activity. In this state, chondrocytes upregulate matrix-degrading enzymes, matrix metalloproteinases (MMPs) and aggrecanases, leading to progressive extracellular matrix breakdown (3, 25). These mechanisms are central to cartilage degeneration in OA and inflammatory joint disease in both humans and horses.

This catabolic shift is consequential as articular cartilage possesses limited intrinsic regenerative capacity due to its avascular nature, low cellular density, and absence of progenitor cell influx following injury (21). Therefore, once structural damage occurs, repair is often incomplete, and degenerative processes may become self-perpetuating. Mechanical instability, synovial inflammation, and sustained cytokine signaling further accelerate cartilage degradation, reinforcing shared pathophysiologic pathways across species and supporting the translational relevance of equine joint research to human disease (26).

### 3.3 Immune cells

Maintenance of joint homeostasis in both humans and horses requires the presence of resident and recruited immune cells that continuously survey the intra-articular environment for signs of injury, infection, or mechanical stress. The synovial membrane is not simply a structural lining but an immunologically active tissue containing macrophages, lymphocytes, dendritic cells, mast cells, and other innate and adaptive immune populations (15, 27). These cells respond to inflammatory mediators, matrix fragments, and PAMPs within the synovium and cartilage, allowing for rapid detection of tissue damage or microbial invasion.

Synovial macrophages are among the most abundant immune cells in the joint and serve as key regulators of inflammation. In both equine and human joints, resident macrophages contribute to debris clearance and immune surveillance under physiologic conditions. However, during OA or septic arthritis, activated macrophages produce IL-1β, TNF-*α*, and IL-6, amplifying local inflammation and stimulating chondrocytes and synoviocytes to release matrix-degrading enzymes (3, 25). Macrophage polarization states (often simplified as pro-inflammatory M1-like and anti-inflammatory M2-like phenotypes) further influence whether inflammation resolves or progresses toward chronic tissue damage (28).

Lymphocytes, particularly T cells, are also present within the synovium and contribute to adaptive immune responses. In human inflammatory arthropathies such as rheumatoid arthritis (RA), T-cell activation plays a central pathogenic role. Although less extensively characterized in horses, lymphocyte infiltration has been documented in equine synovitis and septic arthritis, suggesting mirrored immune mechanisms across species (3, 15). B cells and plasma cells may also contribute through antibody production and cytokine secretion in chronic inflammatory states.

Dendritic cells function as antigen-presenting cells that bridge innate and adaptive immunity. By processing and presenting antigens to T lymphocytes, they help orchestrate targeted immune responses within the joint. In foal and pediatric septic arthritis, rapid recruitment of neutrophils and monocytes to the synovial fluid is critical for bacterial clearance; however, excessive neutrophil activation can exacerbate cartilage injury through proteolytic enzymes and reactive oxygen species (3, 9).

**Figure 1:**
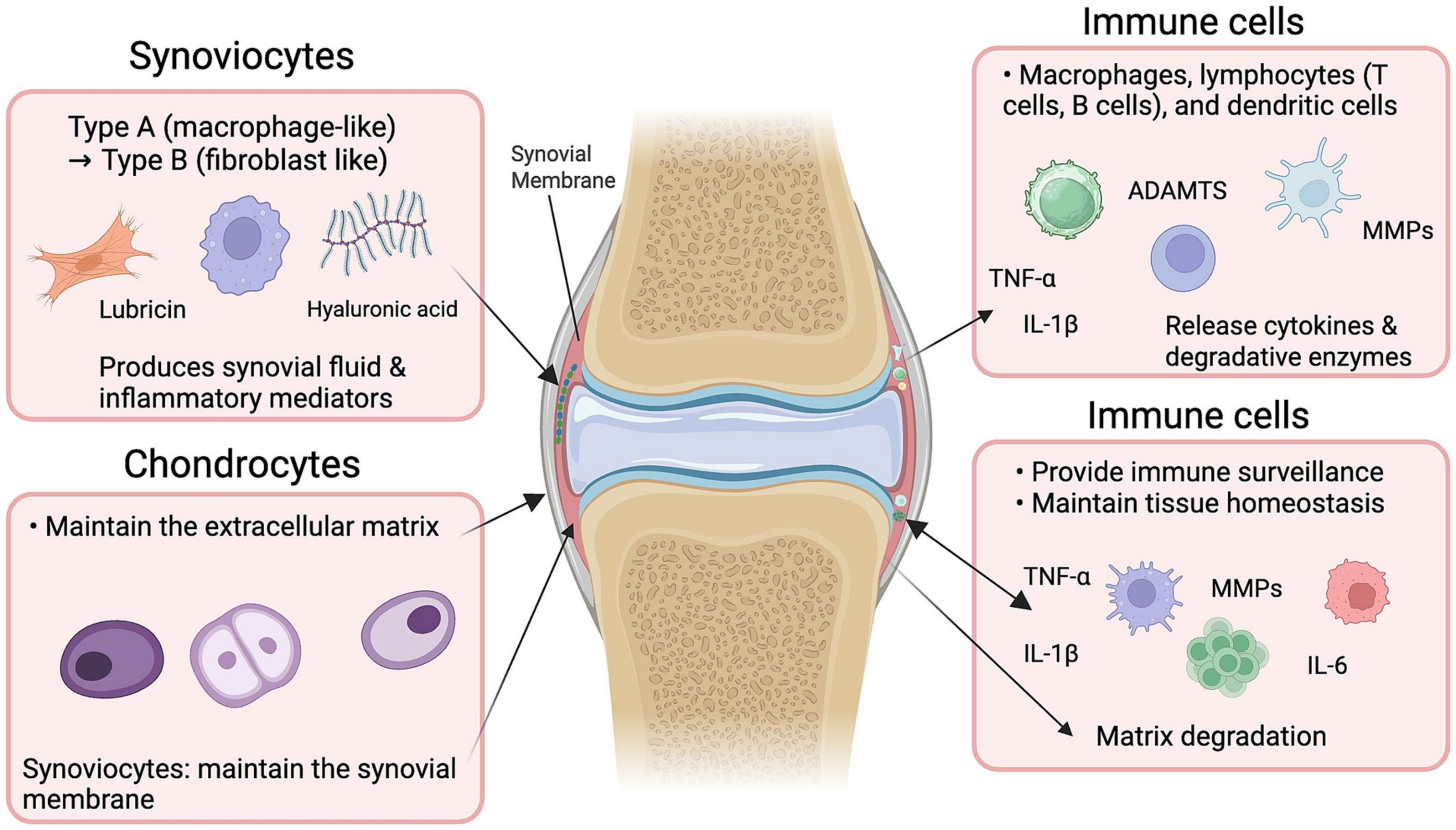
Overview of the major cellular populations within the synovial joint and their roles in maintaining joint homeostasis.

Under normal conditions, these immune cells operate in a tightly regulated balance that supports tissue repair and resolution of inflammation. Disruption of this equilibrium, whether due to repetitive mechanical overload, age-related degeneration, or bacterial infection can shift the joint environment toward persistent inflammation and structural deterioration (Figure 1). The similarities in immune cell composition and inflammatory signaling pathways between equine and human joints further support the translational relevance of comparative joint research.

### 3.4 Cytokine signaling pathways relevant to joint disease

Cytokine-mediated signaling pathways are integral to the development and progression of joint disease, regulating inflammatory responses and tissue remodeling within the synovium, cartilage, and subchondral bone. Key pro-inflammatory cytokines, TNF-*α*, IL-1β, and IL-6, activate NF-κB and JAK/STAT intracellular pathways, which drive expression of inflammatory mediators, recruit immune cells, and promote matrix degradation (29). The NF-κB pathway functions as a major regulator of inflammatory and immune responses in both equine and human joints. Activation occurs in response to stimuli such as pathogen-associated or damage-associated molecular patterns (PAMPs and DAMPs), pro-inflammatory cytokines, and mechanical stress. Engagement of pattern-recognition receptors activates the IκB kinase complex, leading to degradation of inhibitory IκB proteins and nuclear translocation of NF-κB. Once activated, NF-κB promotes transcription of cytokines, chemokines, adhesion molecules, nitric oxide synthase, and matrix-degrading enzymes, shifting the joint environment toward a catabolic state (26). Chronic NF-κB activation contributes to synovial inflammation and cartilage degradation in OA, while acute activation during septic arthritis drives rapid cytokine release and neutrophil recruitment that accelerates tissue damage.

The JAK/STAT pathway represents another key cytokine-responsive signaling mechanism within joint tissues. Cytokine binding to type I or II receptors activates associated Janus kinases, leading to phosphorylation and dimerization of STAT transcription factors, which then induces transcription of genes involved in inflammation, immune cell survival, and tissue remodeling (30). In synoviocytes, chondrocytes, and immune cells, inflammatory cytokines and interferons activate JAK/STAT signaling, contributing to synovial hyperplasia, immune cell persistence, and cartilage matrix breakdown. Persistent activation is particularly prominent in RA, where IL-6 mediated STAT3 signaling promotes pannus formation and progressive joint destruction (31). Increasing evidence also implicates JAK/STAT signaling in OA through IL-6 driven expression of catabolic enzymes such as MMP-13 and ADAMTS-5 (32). As NF-κB and JAK/STAT pathways are highly conserved across species, studies in equine joint disease provide important translational insight into human inflammatory arthritis (Table 1). Targeting these pathways, particularly through JAK inhibitors already used in human medicine, represents a promising strategy for modulating cytokine-driven inflammation and limiting structural joint damage across species.

**Table 1:** Major cytokine-mediated signaling pathways involved in inflammatory joint disease.

Feature	NF-κB signaling pathway	JAK/STAT signaling pathway
Primary activation stimuli	TNF-*α*; IL-1β; PAMPs; DAMPs; mechanical stress; microbial components	IL-6, interferons, and γ-chain cytokines
Key receptors/sensors	Pattern-recognition receptors (e.g., Toll-like receptors) and cytokine receptors	Type I and Type II cytokine receptors
Major intracellular components	IκB proteins; IκB kinase (IKK) complex (IKKα, IKKβ, IKKγ/NEMO); NF-κB transcription factors	Janus kinases (JAK1, JAK2, JAK3, TYK2); STAT transcription factors (STAT1–STAT6)
Activation mechanism	IKK phosphorylates IκB → IκB degradation → NF-κB translocates to nucleus → inflammatory gene transcription	Cytokine binding → receptor dimerization → JAK activation → STAT phosphorylation → STAT dimerization and nuclear translocation
Major downstream gene targets	TNF-α, IL-1β, IL-6, chemokines, adhesion molecules; inducible nitric oxide synthase; matrix metalloproteinases (MMPs)	Inflammatory mediators; chemokines; anti-apoptotic proteins; angiogenic factors; matrix-degrading enzymes
Primary cellular targets in joint	Synoviocytes; chondrocytes; macrophages; infiltrating immune cells	Synoviocytes; chondrocytes; macrophages; lymphocytes
Role in joint pathology	Amplifies inflammatory signaling, leukocyte recruitment, and matrix degradation	Links cytokine signaling to transcriptional programs driving inflammation, synovial hyperplasia, and cartilage degradation
Disease context	Chronic activation in OA; acute activation in septic arthritis	Prominent in RA; contributes to inflammatory mechanisms in OA and septic arthritis
Regulatory mechanisms	Controlled by inhibitory IκB proteins	Negative feedback via Suppressors of Cytokine Signaling (SOCS) proteins and Protein Inhibitors of Activated STATs (PIAS) regulators
Therapeutic targeting	Experimental strategies aimed at modulating NF-κB activation	Clinically targeted by JAK inhibitors (e.g., tofacitinib, baricitinib, upadacitinib)

## 4 Key cytokines in equine and human joint disease

### 4.1 Interleukin-1B

Interleukin-1β (IL-1β) is a key mediator of synovial inflammation in septic arthritis and other inflammatory joint diseases (33). Produced primarily by activated macrophages and synoviocytes, IL-1β initiates leukocyte recruitment, upregulation of adhesion molecules, and production of matrix-degrading enzymes matrix metalloproteinases (MMPs) and aggrecanases. In addition to its well-recognized catabolic effects on cartilage, IL-1β increases paracellular permeability and alters ion transport, thereby sustaining inflammatory signaling and joint effusion. Elevated IL-1β concentrations are consistently detected in septic equine synovial fluid and correlate with disease severity (4). Experimental studies demonstrate that IL-1β stimulation also increases mediators such as hyaluronan, MMP-9, and prostaglandin E2, contributing to the degradative environment of septic joints (34). IL-1β is a key mediator in human OA pathogenesis by promoting chondrocyte catabolism, as it induces extracellular matrix degradation through upregulation of MMP’s and suppression of collagen synthesis. Despite its established role in joint inflammation, the specific effects of IL-1β on synovial barrier function and electrophysiologic properties remain poorly characterized in equine tissue, highlighting an important area for future investigation.

### 4.2 Tumor necrosis factor-*α* (TNF-α)

Tumor necrosis factor-α (TNF-α) is a major upstream cytokine driving inflammatory signaling and matrix degradation in joint disease. Produced by macrophages, synovial fibroblasts, and infiltrating immune cells, TNF-*α* activates intracellular pathways NF-κB and MAPKs, leading to transcription of matrix-degrading enzymes and inflammatory mediators (35, 36). In RA, TNF-*α* strongly stimulates fibroblast-like synoviocytes to produce MMP-1, MMP-3, and MMP-13, degrading type II collagen and other extracellular matrix components. TNF-α acts synergistically with IL-1β and IL-6, further accelerating cartilage destruction through amplifying inflammatory signaling and proteolytic cascades (37).

Although traditionally associated with autoimmune arthritis, TNF-*α* also contributes to OA by promoting a catabolic phenotype in chondrocytes and suppressing synthesis of structural matrix components. The clinical efficacy of TNF inhibitors in inflammatory arthritis further emphasizes the dominate factor of TNF-*α*–mediated pathways in linking synovial inflammation to structural joint damage. Importantly, while RA and septic arthritis differ fundamentally in pathogenesis (autoimmune versus infectious), TNF-α is also elevated in septic joints and contributes to acute inflammatory responses and cartilage degradation during infection; however, its precise regulatory role and therapeutic relevance in septic arthritis remain poorly characterized, representing an important area for further investigation.

### 4.3 Interleukin-6 (IL-6)

Interleukin-6 (IL-6) is a pleiotropic cytokine that bridges local synovial inflammation with systemic immune responses. Produced by synovial fibroblasts, macrophages, endothelial cells, and chondrocytes, IL-6 signals through IL-6R and gp130 to activate the JAK/STAT3 pathway (38, 39). Within the joint, IL-6 promotes synovial hyperplasia, leukocyte recruitment, and osteoclast differentiation, contributing to chronic synovitis and tissue remodeling. A distinguishing feature of IL-6 is its role in systemic inflammation. It is the principal inducer of the hepatic acute-phase response, stimulating production C-reactive protein and serum amyloid A, both of which reflect disease activity in inflammatory arthritis (40). IL-6 trans-signaling further expands inflammatory signaling by enabling activation of cells lacking membrane-bound IL-6 receptors, thereby sustaining leukocyte recruitment and synovial inflammation. Therapeutic blockade of IL-6 signaling, with IL-6 receptor inhibitors, significantly reduces inflammatory markers and clinical symptoms in RA, highlighting its central role in both local and systemic disease processes.

### 4.4 Interleukin-10 (IL-10)

Interleukin-10 (IL-10) functions as a key anti-inflammatory cytokine that limits excessive immune activation within the joint. Produced by regulatory T cells, macrophages, dendritic cells, and B cells, IL-10 counteracts the effects of pro-inflammatory cytokines TNF-*α*, IL-1β, and IL-6 (41, 42). Through activation of JAK1/TYK2 and STAT3 signaling, IL-10 induces expression of suppressor molecules that inhibit NF-κB activity and reduce production of inflammatory cytokines, chemokines, and matrix-degrading enzymes. Within the synovium, IL-10 suppresses macrophage cytokine release, reduces antigen presentation, and decreases MMP production by synovial fibroblasts, thereby helping preserve extracellular matrix integrity (Table 2). However, in chronic inflammatory arthritis, endogenous IL-10 responses are often insufficient to counterbalance dominant pro-inflammatory pathways. This imbalance highlights the importance of having multiple anti-inflammatory cytokines to maintain immune homeostasis and suggests potential therapeutic strategies aimed at enhancing IL-10 mediated along with additional anti-inflammatory signaling.

**Table 2:** Major cytokines involved in inflammatory joint disease and their mechanisms of action in the joint.

Component	IL-1β	TNF-α	IL-6	IL-10
Primary cellular sources	Activated macrophages; synoviocytes	Macrophages; fibroblast-like synoviocytes; infiltrating immune cells	Synovial fibroblasts; macrophages; endothelial cells; chondrocytes	Regulatory T cells; macrophages; dendritic cells; B cells
Major signaling pathways	NF-κB; MAPK	NF-κB; MAPK (ERK, p38, JNK); JAK/STAT cross-talk	JAK1/JAK2 → STAT3	JAK1/TYK2 → STAT3
Major effects in the joint	Promotes leukocyte recruitment; induces MMPs and aggrecanases; increases synovial permeability; disrupts synovial barrier function	Induces MMP-1, MMP-3, and MMP-13; stimulates inflammatory cytokine production; suppresses cartilage matrix synthesis	Promotes synovial hyperplasia; leukocyte recruitment; osteoclast differentiation	Suppresses inflammatory cytokine production; inhibits MMP expression; reduces antigen presentation
Role in joint pathology	Key mediator of synovial inflammation and cartilage catabolism, particularly in septic arthritis	Central upstream driver of inflammatory signaling and matrix degradation in RA and OA	Links local synovial inflammation with systemic acute phase responses	Anti-inflammatory regulator that restrains synovial inflammation and tissue damage
Systemic effects	Contributes to inflammatory mediator production within joints	Sustains inflammatory cytokine networks	Major inducer of hepatic acute phase proteins (CRP, SAA, fibrinogen); contributes to fever and anemia of chronic disease	Limits excessive systemic inflammatory responses
Therapeutic relevance	Target of IL-1 inhibitors in inflammatory disease	Target of TNF inhibitors (e.g., infliximab, etanercept, adalimumab)	Target of IL-6 pathway inhibitors (e.g., tocilizumab, sarilumab)	Experimental therapeutic target aimed at enhancing anti-inflammatory regulation

## 5 Cytokine profiles in specific joint conditions

### 5.1 Osteoarthritis (OA)

OA is now recognized as a whole-joint disease characterized not only by mechanical cartilage degeneration but also by chronic, low-grade inflammation. In addition to cartilage, the synovium, subchondral bone, and infrapatellar fat pad contribute to a sustained inflammatory microenvironment marked by modest yet persistent elevations in cytokines, chemokines, and matrix-degrading enzymes that disrupt joint homeostasis (43, 44).

A central feature of OA pathophysiology is cytokine imbalance. Low-level production of TNF-*α*, IL-1β, and IL-6, by synovial macrophages and activated chondrocytes stimulates matrix metalloproteinases and aggrecanases ADAMTS-4 and ADAMTS-5. These enzymes degrade type II collagen and aggrecan, the principal structural components of articular cartilage, while simultaneously suppressing anabolic pathways responsible for matrix repair (25, 43). Over time, this imbalance between catabolic and reparative processes leads to progressive cartilage thinning and structural degeneration.

Synovitis further reinforces the inflammatory state. Even though OA synovial inflammation is generally milder than in inflammatory arthritis, macrophage activation and low-level lymphocyte infiltration are common. Cytokines released from synovial tissue diffuse into cartilage and activate chondrocytes, while cartilage degradation products act as DAMPs that stimulate innate immune receptors on synoviocytes and macrophages. The bidirectional signaling establishes a self-perpetuating cycle of inflammation and matrix degradation (44).

IL-6 appears particularly important in maintaining the chronic inflammatory environment in OA through activation of JAK/STAT3 signaling and induction of the catabolic mediator MMP-13. At the same time, present anti-inflammatory cytokines are often insufficient to counterbalance persistent pro-inflammatory signaling. This relative deficiency of regulatory mediators contributes to a state of cytokine disequilibrium in which modest but sustained inflammatory signaling gradually drives structural damage (25).

Metabolic factors can also influence OA inflammation. Leptin, adiponectin, and resistin produced by adipose tissue and the infrapatellar fat pad modulate cytokine production and matrix degradation. Consequently, obesity-associated systemic inflammation may exacerbate local joint pathology independent of mechanical loading, supporting the concept that OA is partly a metabolically influenced inflammatory disorder rather than purely a “wear-and-tear” condition (43). Overall, OA represents a chronic state of low-grade inflammatory dysregulation in which persistent cytokine imbalance gradually erodes cartilage and alters subchondral bone architecture. Recognizing this inflammatory component has important implications for developing disease-modifying therapies aimed at restoring joint homeostasis.

### 5.2 Septic arthritis

Septic arthritis represents the most acute and destructive form of inflammatory joint disease and is characterized by rapid-onset synovitis driven by massive cytokine release and neutrophil recruitment. The condition typically arises from direct microbial invasion of the synovial space, most commonly by bacterial pathogens such as *Staphylococcus aureus*. Recognition of PAMPs by Toll-like receptors on synovial macrophages and fibroblast-like synoviocytes triggers rapid production of pro-inflammatory cytokines including TNF-*α*, IL-1β, and IL-6, as well as chemokines IL-8 (CXCL8) (45, 46).

This cytokine surge activates the NF-κB and MAPKs pathways, amplifying inflammatory mediator production and promoting expression of matrix-degrading enzymes. IL-6 simultaneously drives the systemic acute-phase response, increasing circulating inflammatory markers such as C-reactive protein. The magnitude and rapid onset of cytokine signaling distinguish septic arthritis from chronic OA or RA (45, 47). A defining feature of septic arthritis is extensive neutrophil recruitment into the joint. Chemokines create strong chemotactic gradients that direct circulating neutrophils across activated synovial endothelium, where adhesion molecules ICAM-1 and VCAM-1 facilitate transmigration (48). Within the joint, neutrophils eliminate pathogens through phagocytosis and release of reactive oxygen species, proteolytic enzymes, and neutrophil extracellular traps. Although these responses are critical for pathogen clearance, they also contribute to collateral damage of cartilage and synovial tissues (46).

Cartilage destruction can occur within days, due to the combined effects of bacterial toxins, host-derived proteases, and cytokine-induced chondrocyte catabolism. TNF-*α* and IL-1β stimulate chondrocytes to produce MMP-1, MMP-3, and MMP-13, while neutrophil-derived enzymes further degrade extracellular matrix components. Increased intra-articular pressure from purulent effusion may also impair cartilage nutrition and viability, accelerating structural damage (45, 47). Although anti-inflammatory cytokines are produced during infection, their regulatory effects are often overwhelmed during the acute phase. While cytokine signaling is essential for microbial clearance in septic arthritis, it is also capable of causing rapid tissue destruction when excessive. Early diagnosis and intervention are therefore critical to limit both infectious burden and cytokine-mediated joint damage.

### 5.3 Synovitis

Synovitis is a central feature of many joint diseases in both humans and horses and is characterized by activation of synovial lining cells, immune cell infiltration, and increased vascular permeability within the synovial membrane (49). The synovium normally consists of a thin intimal layer containing macrophage-like synoviocytes (type A cells) and fibroblast-like synoviocytes (type B cells), supported by a vascularized sub-lining layer. Under physiological conditions, this structure maintains joint homeostasis by regulating synovial fluid composition and providing a selective barrier between the circulation and joint cavity (36, 44). Comparable organization and function are present in equine joints, where synovial integrity is essential for maintaining high-load athletic performance (15, 50).

Early synovitis occurs when synovial macrophages and fibroblast-like synoviocytes respond to mechanical stress, cartilage degradation products, DAMPs, or microbial stimuli through pattern-recognition receptors. Activation of these receptors stimulates NF-κB and JAK/STAT, resulting in production of TNF-*α*, IL-1β, and IL-6 (36, 44). Similar cytokine profiles have been documented in equine OA, indicating conserved inflammatory mechanisms across species (15, 51).

One of the earliest physiologic consequences of synovial inflammation is increased vascular permeability. Histamine, prostaglandins, bradykinin, and vascular endothelial growth factor induce endothelial activation and disruption of intercellular junctions, leading to plasma protein leakage and joint effusion (52). In equine synovitis, increased synovial fluid protein concentrations and leukocyte counts are early diagnostic indicators of this vascular leakage (50). Angiogenesis also plays a significant role in sustaining synovial inflammation. In RA, expansion of the synovial lining and pannus formation require increased vascular supply, and newly formed vessels facilitate continued recruitment of inflammatory cells (52, 53). Although synovial angiogenesis is typically less pronounced in OA, it can still support chronic inflammation and may contribute to pain through neurovascular interactions (15, 44).

Chemokines further regulate immune cell trafficking within inflamed joints. These signaling molecules guide leukocyte migration, promote cytokine production, and influence angiogenesis through interactions with G-protein–coupled chemokine receptors (54, 55). Dysregulated chemokine signaling contributes to persistent synovial inflammation and immune cell recruitment in diseases such as RA (56). Importantly, synovial inflammation is not merely a secondary response to cartilage damage but can actively drive disease progression. Macrophage density within the synovial lining correlates with disease severity and therapeutic response in both human RA and equine OA (36, 51). Persistent synovial inflammation alters synovial fluid composition, impairs nutrient diffusion to avascular cartilage, and promotes progressive structural degeneration. In summary, synovitis involves coordinated activation of cytokine signaling, endothelial permeability, leukocyte recruitment, and angiogenesis. While initially protective, these processes become maladaptive when sustained, creating a chronic inflammatory microenvironment that contributes to cartilage degradation and joint damage (Table 3).

**Table 3:** Cytokine-mediated inflammatory mechanisms in osteoarthritis, synovitis, and septic arthritis.

Component	Osteoarthritis	Synovitis	Septic arthritis
Trigger	Mechanical stress; cartilage degeneration; metabolic factors; DAMPs	Mechanical injury; DAMPs; immune activation; infection	Microbial invasion of the joint (e.g., *Staphylococcus aureus)*
Cytokines/chemokines	TNF-α, IL-1β, IL-6 (low-moderate levels)	TNF-α, IL-1*β*, IL-6, chemokines (moderate levels)	TNF-α, IL-1β, IL-6, IL-8 (CXCL8); very high levels (“cytokine storm”)
Immune cells	Synovial macrophages; Activated chondrocytes	Macrophages; fibroblast-like synoviocytes (FLS); lymphocytes	Neutrophils (dominant); macrophages
Synovial changes	Mild synovitis; mild angiogenesis; low-level vascular permeability	Synovial lining expansion; increased vascular permeability; angiogenesis	Severe synovitis; purulent effusion; endothelial activation
Matrix degradation	↑ MMPs and ADAMTS-4/5; decreased collagen II and aggrecan synthesis	↑ MMPs (MMP-1, MMP-3, MMP-13); cytokine-driven matrix degradation	Neutrophil enzymes; reactive oxygen species (ROS); bacterial toxins; ↑ MMPs
Signaling pathways	NF-κB → JAK/STAT3	NF-κB → JAK/STAT	NF-κB → MAPK
Clinical course	Chronic, slowly progressive disease; gradual cartilage loss	Early inflammatory stage that may precede OA or RA	Acute onset; rapid cartilage destruction and joint damage
Outcome	Chronic cartilage thinning and joint degeneration	Inflammation-driven cartilage degradation	Rapid cartilage destruction and severe joint damage

### 5.4 Comparative cytokine expression in naturally occurring vs. experimental models

Comparative evaluation of cytokine expression in equine and human joint disease reveals substantial molecular overlap alongside important species-specific differences. Horses develop naturally occurring OA and septic arthritis that closely resemble human disease in clinical presentation, histopathology, and inflammatory signaling, supporting their value as a translational large-animal model (3, 57). Because equine OA frequently follows athletic injury and repetitive biomechanical stress, it parallels post-traumatic OA in humans more closely than many small-animal models.

In both equine and human OA, synovial fluid and synovium demonstrate persistent, low-grade elevation of TNF-*α*, IL-1β, and IL-6 (11, 58). These mediators promote MMP production, particularly MMP-13, which drives progressive cartilage degradation (25). Macrophage activation and fibroblast-like synoviocyte proliferation are also conserved features across species (44). Although present, anti-inflammatory IL-10 is detectable, its regulatory effects are insufficient to counter ongoing catabolic signaling, resulting in sustained inflammatory disequilibrium.

Differences between species are observed primarily in magnitude and kinetics of cytokine responses rather than the identity of the mediators involved. Acute post-traumatic equine OA may exhibit higher early IL-1β concentrations compared with typical idiopathic human OA, reflecting the injury-driven nature of disease in athletic horses (59). In contrast, human OA is generally characterized by more sustained, lower-grade elevations of pro-inflammatory cytokines over time. Thus, the cytokine profile is considered conserved, as the same key inflammatory mediators are present across species; however, the initiating triggers, intensity of response, and temporal dynamics of cytokine expression differ. Septic arthritis in both species is characterized by rapid, high-amplitude increases in TNF-*α*, IL-1β, IL-6, and neutrophil chemoattractant IL-8 (CXCL8), with marked neutrophil predominance in synovial fluid within hours of infection (47, 60). Equine septic arthritis closely mirrors human bacterial arthritis, particularly with respect to IL-6 elevation and neutrophil recruitment, reinforcing the horse as a relevant model for acute cytokine-mediated joint inflammation.

Experimentally induced synovitis models, including intra-articular lipopolysaccharide (LPS) injection in horses, produce rapid and synchronized cytokine surges, with early TNF-*α* and IL-1β peaks followed by secondary IL-6 elevation (61). Compared with naturally occurring OA, experimentally induced synovitis models typically generate rapid, high-amplitude cytokine responses that reflect acute inflammation rather than the chronic low-grade inflammatory state observed in spontaneous disease. Overall, equine joint disease shares key conserved inflammatory pathways with human pathology, including TNF-α and IL-1β driven MMP induction, IL-6 mediated JAK/STAT signaling, and chemokine-directed neutrophil recruitment in septic arthritis (11, 44). The principal divergence lies in cytokine magnitude, timing, and regulatory balance rather than in the specific mediators expressed.

The horse therefore represents a valuable translational bridge: it develops naturally occurring OA with overlapping molecular and cellular mechanisms comparable to human disease, experiences clinically relevant septic arthritis, and allows repeated synovial sampling and detailed physiologic assessment due to joint size. While differences in inflammatory dynamics should be considered when concluding direct comparisons, comparative cytokine profiling across species enhances mechanistic understanding and supports the translational development of cytokine-targeted therapies.

## 6 Diagnostic and prognostic applications

### 6.1 Synovial fluid cytokine profiling

Synovial fluid cytokine profiling provides a direct assessment of the intra-articular inflammatory environment and offers diagnostic and prognostic value in both equine and human joint disease (11, 44). As synovial fluid reflects local pathology more accurately than systemic biomarkers, measurement of inflammatory mediators can improve disease classification and monitoring across species.

Cytokine concentrations help differentiate septic arthritis from non-infectious joint disease. Acute septic arthritis is characterized by marked elevations in TNF-*α*, IL-1β, IL-6, and neutrophil-associated chemokine IL-8 (CXCL8), accompanied by neutrophilic leukocytosis and increased synovial fluid protein (47, 60). IL-6 is particularly informative because of its rapid and high-amplitude increase during infection. Similar cytokine profiles occur in equine septic synovitis, where elevated IL-1β and TNF-*α* correlate with neutrophil predominance and disease severity (59). In contrast, OA in both species exhibits persistent but lower-magnitude increases in IL-1β, TNF-*α*, and IL-6, reflecting chronic low-grade inflammation rather than the acute cytokine surge seen in infection (3, 11). In RA, synovial fluid typically contains elevated IL-6, TNF-*α*, IL-17, and T-cell associated chemokines, although horses do not develop classic autoimmune RA (36).

Cytokine concentrations may also provide prognostic information. In human inflammatory arthritis, elevated IL-6 and TNF-α correlate with radiographic progression and matrix metalloproteinase mediated cartilage degradation, particularly MMP-13 (11). Similarly, increased IL-1β and TNF-*α* following joint injury in horses are associated with subsequent cartilage deterioration (3). Experimental synovitis models further demonstrate that the magnitude of early cytokine release may predict later structural change (62). Cytokine profiling is also useful for monitoring treatment response. In equine medicine, intra-articular corticosteroids and orthobiologic therapies such as autologous conditioned serum have been evaluated by monitoring changes in IL-1β and TNF-*α* concentrations (63).

### 6.2 Biomarker potential for early detection and monitoring

Early identification of joint disease is a priority in both equine and human medicine because structural cartilage damage is largely irreversible (3, 11). Synovial cytokines have therefore been investigated as biomarkers of early intra-articular inflammation that may precede detectable structural change. Across species, early joint injury is associated with synovial activation and increased IL-1β, TNF-*α*, and IL-6 before overt cartilage deterioration occurs (44). In equine athletes, post-traumatic synovitis produces elevations in IL-1β and TNF-*α* within days of injury, even when imaging findings remain minimal (59). Similarly, after acute anterior cruciate ligament injury in humans, synovial IL-6, IL-1β, and TNF-α increase rapidly, implicating conserved inflammatory pathways in post-traumatic OA development (64).

IL-6 is particularly important because of its role in JAK/STAT signaling and its downstream catabolic effects in chondrocytes (11). Persistent elevations of IL-1β, TNF-α, and IL-6 correlate with MMP activation, especially MMP-13, and progressive cartilage degradation in both species. In equine OA, sustained cytokine elevation after injury is associated with ongoing degenerative change, whereas declining concentrations may indicate resolution of acute synovitis (3).

Multi-marker biomarker panels may further improve predictive accuracy. Combining inflammatory cytokines with cartilage turnover markers, such as type II collagen degradation fragments or aggrecan neoepitopes, improves correlation with structural progression in human OA, with similar approaches under investigation in equine research (65). Regulatory imbalance may also be informative, as inadequate IL-10 relative to pro-inflammatory cytokines may indicate failure of inflammatory resolution (44). Clinical application remains limited by variability in sampling timing, joint type, exercise status in horses, and assay standardization (59). Establishing species-specific reference ranges will be essential for broader implementation.

### 6.3 Correlation with clinical severity and imaging findings

For synovial biomarkers to have clinical value, they must correlate with disease severity and structural progression. Evidence from both equine and human studies supports associations between pro-inflammatory cytokines and clinical outcomes. In equine OA, synovial IL-1β, TNF-*α*, and prostaglandin E₂ are elevated compared with healthy joints and correlate with lameness severity and joint effusion (3, 61). In human knee OA, synovial IL-6 and TNF-α correlate with pain severity and functional impairment and are associated with symptomatic progression (66, 67). In septic arthritis, markedly elevated IL-6 and IL-1β distinguish infectious from non-infectious arthritis and reflect inflammatory severity in both species (59, 68).

Imaging provides structural assessment, whereas synovial biomarkers reflect molecular activity. Correlating these domains improves disease staging and monitoring. In equine OA, elevated IL-1β and PGE₂ are associated with radiographic osteophytes and subchondral bone sclerosis (3). In humans, synovial IL-6 and MMP concentrations correlate with MRI-detected synovitis, cartilage loss, and bone marrow lesions (69, 70). Longitudinal studies further show that elevated baseline cytokine levels predict future radiographic progression (71). Together, these findings demonstrate that synovial cytokines reflect active molecular inflammation and correlate with both clinical severity and structural damage. Due to molecular changes preceding radiographic abnormalities, cytokine profiling may help identify early disease and guide monitoring strategies.

## 7 Therapeutic modulation of synovial cytokines

### 7.1 Biologic therapies

Targeted biologic therapies have transformed management of human inflammatory arthritis, particularly RA. Monoclonal antibodies and receptor fusion proteins targeting TNF-*α*, specifically, infliximab, adalimumab, and etanercept, reduce synovial inflammation and slow structural progression by blocking downstream inflammatory signaling (71). IL-6 receptor inhibitors, including tocilizumab and sarilumab, suppress JAK/STAT signaling and rapidly decrease systemic inflammatory markers (40).

In contrast, equine joint disease management typically relies on local therapies rather than systemic biologics. Treatments commonly include intra-articular corticosteroids, hyaluronan, and orthobiologic approaches. A notable translational parallel is inhibition of IL-1 signaling through autologous conditioned serum (ACS), which increases intra-articular IL-1 receptor antagonist (IL-1Ra) and counteracts IL-1β mediated inflammation (72). Experimental gene therapy delivering IL-1Ra has also demonstrated reduced synovial inflammation and cartilage damage in equine models (73).

### 7.2 Gene therapy

Gene therapy targeting IL-1 receptor antagonist (IL-1Ra) represents a promising strategy for sustained modulation of intra-articular inflammation. Because IL-1β drives synovial inflammation and cartilage degradation in both species, increasing local IL-1Ra production may restore cytokine balance within the joint (3, 11).

Early human studies demonstrated feasibility using retrovirally modified synovial fibroblasts expressing IL-1Ra (74). More recently, adeno-associated viral vectors have shown potential for sustained gene expression with favorable safety profiles (75). Equine experimental OA studies using adenoviral or AAV vectors encoding IL-1Ra have demonstrated decreased synovial inflammation and cartilage degradation, providing valuable translational insight. Although challenges remain, including vector delivery, immune responses, and regulatory constraints, gene therapy highlights the potential for modifying the intra-articular cytokine environment to influence disease progression rather than only symptoms.

### 7.3 Regenerative medicine: mesenchymal stromal cells

Mesenchymal stromal cells (MSCs) are increasingly used in both human and equine joint disease primarily for their immunomodulatory effects rather than direct cartilage regeneration (76). MSCs secrete anti-inflammatory cytokines, growth factors, and extracellular vesicles that reduce inflammatory signaling and modulate immune cell activity (77).

In human OA, MSC therapy reduces IL-1*β*, TNF-*α*, and IL-6 while increasing anti-inflammatory mediators IL-10 and TGF-β. Similar effects have been observed in equine studies, where MSCs suppress inflammatory signaling in synoviocytes and chondrocytes and reduce MMP expression (78, 79). Although clinical improvements in pain and function are frequently reported, long-term structural cartilage regeneration remains inconsistent.

### 7.4 Hemoperfusion and extracorporeal cytokine removal

Extracorporeal blood purification techniques such as hemoperfusion aim to reduce circulating cytokines during severe systemic inflammation. These approaches are primarily used in human critical care for sepsis and septic shock, where excessive cytokine production contributes to organ dysfunction (80, 81).

Although their role in isolated joint disease is limited, cytokine adsorption may theoretically benefit cases of septic arthritis complicated by systemic inflammatory response syndrome. In equine medicine, extracorporeal cytokine removal is not routinely used, though hemoperfusion and plasma exchange have been explored in endotoxemia and systemic sepsis (82). Management of equine septic arthritis remains focused on joint lavage, antimicrobial therapy, and intra-articular anti-inflammatory treatment (83) (Table 4).

**Table 4:** Comparative human-equine insights into synovial cytokines.

Component	Human	Equine
Diagnostic cytokine profiles	IL-6, IL-1β, TNF-α, and IL-8 differentiate septic arthritis from OA; IL-6 shows strong discriminatory performance.	IL-6, IL-1β, TNF-α, and IL-8 distinguish septic synovitis from OA; elevations correlate with neutrophil predominance and clinical severity.
Chronic OA cytokine patterns	Low-grade but persistent IL-1β, IL-6, TNF-α; cytokine imbalance rather than acute surges.	Mirrors human chronic OA profile; naturally occurring OA shows persistent pro-inflammatory cytokines.
Early disease biomarkers	Post-ACL injury: rapid IL-6, IL-1β, TNF-α increases precede radiographic OA; multi-marker panels improve prediction.	Early joint injury: rapid increases in IL-1β/TNF-α even when imaging is normal; cytokine peaks predict later degeneration.
Clinical severity correlation	IL-6 and TNF-α correlate with pain, WOMAC scores, and MRI synovitis.	IL-1β, TNF-α, PGE₂ correlate with lameness and effusion; experimental synovitis parallels clinical scores.
Imaging associations	Cytokines correlate with MRI synovitis, cartilage loss, and bone marrow lesions; predict joint space narrowing.	IL-1β and PGE₂ associated with osteophytes and subchondral sclerosis on radiographs.
Biologic therapies	TNF-α inhibitors, IL-6 receptor blockers, IL-1 blockade, B-cell depletion, T-cell costimulation inhibitors validated in RA.	No licensed monoclonal antibodies; intra-articular corticosteroids, hyaluronan, ACS, and PRP predominating.
Gene therapy	AAV-IL-1Ra reduces synovitis in preclinical models; early retroviral IL-1Ra trials showed safety.	Equine adenoviral/AAV-IL-1Ra reduces synovitis, cartilage fibrillation, and catabolic mediators; key translational data.
MSC immunomodulation	MSCs reduce IL-1β, TNF-α, IL-6; promote IL-10/TGF-β; symptom improvement > structural change.	MSCs suppress IL-1β/TNF-α signaling and MMP-13/ADAMTS; widely used clinically in athletes.
Extracorporeal therapies	Hemoperfusion used in systemic sepsis; reduces circulating IL-6, IL-1β, TNF-α.	Rare/Investigational; considered in endotoxemia and severe SIRS; septic arthritis managed locally.

## 8 Challenges and future directions

Advancing translational research between equine and human joint disease requires addressing several methodological and clinical gaps. A primary limitation is the availability of validated, species-specific cytokine assays. In human medicine, standardized multiplex immunoassays and ELISAs provide reproducible cytokine quantification with established reference ranges (11). In contrast, equine cytokine analysis is often limited by antibody cross-reactivity, incomplete assay validation, and small cohort sizes (84, 85). The use of human-based kits may further compromise accuracy due to interspecies protein sequence differences. While the development of equine-specific multiplex platforms and standardized sampling protocols will improve reproducibility and facilitate cross species comparisons, interpretation of synovial cytokine data remains inherently complex. This is due to substantial variability in sampling timing, disease stage, joint type, and assay methodology, all of which can significantly influence cytokine profiles.

Human rheumatology benefits from large longitudinal cohorts linking cytokine profiles with imaging progression and clinical outcomes (71). These studies have identified prognostic biomarkers and predictors of therapeutic response. In equine medicine, many investigations rely on short-term or experimentally induced synovitis models (3). While mechanistically informative, these models may not fully reflect chronic, naturally occurring OA. Longitudinal equine studies incorporating serial synovial sampling, advanced imaging, and standardized lameness assessment would strengthen translational relevance.

High-throughput technologies offer systems-level insight beyond individual cytokines. In human OA and RA, transcriptomic profiling of synovial tissue has identified inflammatory “endotypes” associated with differential therapeutic response (86). Applying transcriptomic, proteomic, and metabolomic analyses in equine joint disease may reveal conserved inflammatory networks and species-specific regulatory pathways, refining therapeutic targeting.

Precision approaches are increasingly used in human rheumatology, where biologic selection is guided by disease phenotype and biomarker profile (69). Expanding cytokine and molecular profiling in equine medicine may allow more tailored intra-articular therapies, such as MSCs or IL-1Ra based products, based on an individual inflammatory signature. Achieving this requires validated biomarkers, standardized outcomes, and cost-effective diagnostics. Bridging the translational gap between equine and human joint disease will require harmonized assay development, expanded longitudinal datasets, and integration of multi-omics technologies. Collaborative cross-species research can clarify conserved inflammatory pathways and accelerate development of mechanism-based, cytokine-guided therapies.

## 9 Conclusion

Cytokines are central regulators of joint inflammation in both horses and humans, orchestrating synovial activation, MMP production, cartilage degradation, and vascular changes (3, 11). Pro-inflammatory mediators IL-1β, TNF-*α*, and IL-6 drive disease progression, whereas IL-10 and IL-1Ra provide counter-regulatory control. In OA, persistent low-grade cytokine imbalance sustains synovitis and structural degeneration, while septic arthritis and acute synovitis are characterized by rapid cytokine upregulation and neutrophil-mediated tissue injury (Table 5) (45). Despite phenotypic differences, most notably the autoimmune component of human RA, the core inflammatory networks show substantial cross-species overlap (69).

**Table 5:** Summary of translational insights and clinical implications of cytokine-mediated joint disease.

Domain	Key conclusions	Translational implication
Core pathophysiology	Cytokines (IL-1β, TNF-*α*, IL-6) are central regulators of synovial inflammation, matrix degradation, and structural joint damage across species.	Shared mechanisms support use of equine disease as a translational model for human joint pathology.
Inflammatory balance	Disease progression reflects imbalance between pro-inflammatory cytokines and anti-inflammatory regulators (e.g., IL-10, IL-1Ra).	Therapies restoring cytokine balance may be more effective than single-target inhibition.
Disease-specific cytokine patterns	OA exhibits chronic low-grade cytokine elevation, whereas septic arthritis and acute synovitis involve rapid, high-amplitude cytokine responses.	Cytokine magnitude and kinetics can guide disease classification and treatment urgency.
Cross-species conversation	Core cytokine networks and signaling pathways (NF-κB, JAK/STAT) are highly conserved between equine and human disease.	Enables bidirectional translation of therapeutics, biomarkers, and mechanistic insights.
Diagnostic applications	Synovial cytokine profiling improves early detection, disease stratification, and prognostication when combined with clinical and imaging data.	Integration of molecular and imaging data enhances precision medicine approaches.
Therapeutic strategies	Cytokine-targeted therapies (biologics in humans; orthobiologics, IL-1Ra, MSCs in horses) demonstrate parallel approaches to modulating inflammation.	Cross-species comparison may accelerate development of targeted intra-articular therapies.
Translational model value	Horses provide a clinically relevant large-animal model with naturally occurring disease and comparable joint structure.	Valuable platform for translational and preclinical therapeutic development.
Current limitations	Key barriers include lack of standardized equine assays, limited longitudinal data, and variability in study design.	Standardization and larger datasets are required to improve reproducibility and clinical adoption.

The comparative evidence reviewed herein demonstrates that cytokine-mediated pathways underlying joint disease are highly conserved between horses and humans, supporting the value of a translational, cross-species approach to understanding joint pathology. Human studies have provided extensive insight into cytokine biology, biomarker development, and targeted therapeutic strategies, whereas equine research offers a clinically relevant large-animal model of naturally occurring joint disease. Together, these complementary perspectives highlight opportunities to accelerate advances in both veterinary and human medicine through greater comparative investigation.

Diagnostic and therapeutic strategies increasingly reflect this molecular framework. Synovial cytokine profiling and integration with advanced imaging have improved disease stratification and prognostication. In human medicine, cytokine-targeted biologics against TNF-*α*, IL-6, and IL-1 have transformed inflammatory arthritis management (69). Orthobiologic therapies, IL-1Ra-based treatments, gene transfer approaches, and mesenchymal stromal cell therapies represent parallel efforts in equine patients to modulate intra-articular cytokine networks (71, 76). Collectively, these advances suggest that early recognition and targeted modulation of synovial inflammation may represent a critical strategy for slowing OA progression and improving long-term outcomes in horses.

Strengthening translational alignment will require standardized equine-specific cytokine assays, longitudinal studies in naturally occurring disease, and harmonized clinical and imaging outcome measures. Owing to comparable joint size, naturally occurring athletic OA, and established experimental models, the horse serves as both a clinical population and a valuable translational platform (3).

Advancing cytokine-directed joint therapeutics will depend on interdisciplinary collaboration and bidirectional translational research. Integrating mechanistic insight with clinical application across species offers the opportunity to shift from symptomatic management toward earlier detection and targeted modulation of inflammatory pathways, ultimately improving long-term joint health in both equine and human patients. Future studies should prioritize approaches that directly link cytokine signaling to functional tissue outcomes, including *ex vivo* membrane physiology models such as using chambers to assess synovial barrier integrity, ion transport, and inflammatory responses under controlled conditions.
