# Supplementary material for: Cytokine-mediated pathophysiology of equine and human joint disease: mechanisms, biomarkers, and therapeutic targets
Source: Front Vet Sci. 2026 Jul 17;13:1886084. doi: 10.3389/fvets.2026.1886084 (PMC13424700; doi:10.3389/fvets.2026.1886084)
Supplement: Supplementary file 1 [file Table_1.docx]

**Supplementary Table 1. Systematic Evidence Matrix of Cytokine-Mediated Joint Disease Studies**

| **Study (Author, Year)** | **Species** | **Condition** | **Model type** | **Cytokines Evaluated** | **Key Findings** | **Clinical/Translational Relevance** |
| --- | --- | --- | --- | --- | --- | --- |
| **Kapoor et al., 2011** | Human | OA | Clinical/Review | IL-1β, TNF-α, IL-6 | Pro-inflammatory cytokines regulate cartilage degradation and synovial inflammation | Supports cytokine-targeted therapies |
| **Mccllwraith et al., 2021** | Equine | OA | Natural | IL-1β, TNF-α | Equine OA mirrors human disease | Translational model |
| **Sanchez-Lopez et al., 2022** | Human | OA | Clinical | IL-6, TNF-α, IL-1β | Synovitis linked to progression | Highlights inflammation role |
| **Watkins et al., 2021** | Equine | Synovitis | Experimental | IL-1β | Induces inflammatory mediators | Model validation |
| **Bertone et al., 2001** | Equine | OA / Synovitis | Clinical | IL-1β, TNF-α | Correlates with lameness | Biomarker utility |
| **Palmer & Bertone, 1994** | Equine | Synovitis | Experimental | TNF-α, IL-1β | Rapid cytokine surge | Mechanistic model |
| **Ross et al., 2012** | Equine | Synovitis | Experimental | IL-1β | Temporal cytokine prediction | Therapeutic evaluation |
| **Matthews et al., 2010** | Human | Septic arthritis | Clinical | IL-6, TNF-α | Drives joint destruction | Diagnostic differentiation |
| **Shirtliff & Mader, 2002** | Human | Septic arthritis | Mechanistic | TNF-α | Inflammation causes damage | Early treatment importance |
| **Scanzello & Goldring 2012** | Human | OA | Mechanistic | IL-1β, TNF-α | Synovitis drives cartilage damage | Therapeutic target |
| **Robinson et al., 2016** | Human | OA | Mechanistic | IL-6 | Low-grade inflammation drives OA | Expands OA paradigm |
| **Kraus et al., 2011** | Human | OA | Biomarker | IL-6 | Correlates with progression | Supports clinical trials |
| **Frisbie et al., 2007** | Equine | OA | Experimental | IL-1β | IL-1Ra reduces inflammation | Orthobiologic basis |
| **Goodrich et al., 2024** | Equine | OA | Gene therapy | IL-1 pathway | Improves outcomes | Novel therapy |
| **Tanaka et al., 2014** | Human | RA/OA | Mechanistic | IL-6 | Drives systemic inflammation | Biologic target |
| **Morris et al., 2018** | Human | Arthritis | Mechanistic | JAK/STAT | Regulates inflammation | JAK inhibitors |
| **Liu et al., 2022** | Human | OA | Mechanistic | TNF-α | Controls degeneration | Therapeutic target |
| **De Grauw et al., 2009** | Equine | Synovitis | Experimental | IL-1β | Sustained cytokine elevation | Early OA model |
| **Fortier & Travis, 2011** | Equine | OA | Clinical | IL-1β | MSC modulates inflammation | Regenerative therapy |
| **Cobalth et al., 2020** | Equine | OA | Clinical | IL-1β | Improves outcomes | Widely used clinically |
| **Ronco & Bellomo, 2022** | Human | Sepsis | Clinical | IL-6 | Reduces cytokines | Extracorporeal therapy |
| **Hobbs et al., 2025** | Equine | Sepsis | Experimental | TNF-α | Modifies response | Translational relevance |
